# Is it a true left bundle branch block or not?

**DOI:** 10.1007/s10840-023-01530-y

**Published:** 2023-03-28

**Authors:** Karol Curila, Pavel Jurak, Mihail G. Chelu, Gaurav Upadhyay, Kamil Sedlacek, Pavel Osmancik

**Affiliations:** 1 Cardiocenter, Third Faculty of Medicine, Charles University and University Hospital Kralovske Vinohrady, Srobarova 50, 100 34 Prague, Czech Republic; 2 Institute of Scientific Instruments, the Czech Academy of Sciences, Brno, Czech Republic; 3 Department of Medicine, Section of Electrophysiology, Baylor College of Medicine, Houston, TX USA; 4 Center for Arrhythmia Care, Section of Cardiology, Department of Medicine, Pritzker School of Medicine, The University of Chicago Medicine, Chicago, IL USA; 5 1St Department of Internal Medicine - Cardiology and Angiology, University Hospital and Charles University Medical Faculty, Hradec Kralove, Czech Republic

Among patients with wide QRS complexes of nonRBBB morphologies on ECG, there are those with preserved left septal Purkinje activation, i.e., intraventricular conduction delay (IVCD) [1]. Unfortunately, twelve lead ECGs do not allow to discriminate them reliably from those with disrupted LBB activation, i.e., trueLBBB.

IVCD and trueLBBB differ in the ventricular activation sequence. While during trueLBBB, LV lateral wall activation is postponed due to the trans-septal conduction delay, IVCDs have various LV activation patterns [2]. Left ventricular activation during trueLBBB can be reproduced by pre-existing the right ventricular (RV) septum with RV septal pacing (RVSP). On the other hand, RV septal pacing in patients with IVCD leads to additional trans-septal conduction delay, which is not present during the spontaneous rhythm [3].

The activation sequence of ventricular segments may be easily visualized using an ultra-high-frequency ECG (UHF-ECG). We hypothesized that by studying UHF-ECG ventricular activation sequences during the spontaneous rhythm and RVSP, we could discriminate between trueLBBB and IVCD and observe different responses to RV septal pacing.

Ventricular depolarization patterns were visualized using UHF-ECG in two patients with heart failure and wide QRS complexes. Patient 1 had a history of transmural myocardial infarction of the anterior wall, left ventricular ejection fraction (LVEF) of 20%, and a spontaneous QRS complex of IVCD morphology with QRSd of 170 ms. Patient 2 had heart failure due to severe aortic regurgitation with an LVEF of 42% and developed a new-onset 1st-degree AV block with LBBB after transfemoral aortic valve insertion. RVSP was performed in both patients using a Select Secure 3830 (Medtronic, USA) lead. The pacing location was just below the level of the tricuspid valve, visualized using contrast or by identifying the position of the His bundle using another 3830 lead—Fig. 1, panels A and D. Both patients signed informed consent, and data for UHF-ECG analyses were collected for 5–7 min of spontaneous rhythms and RVSP during an implant procedure.

**Fig. 1:**
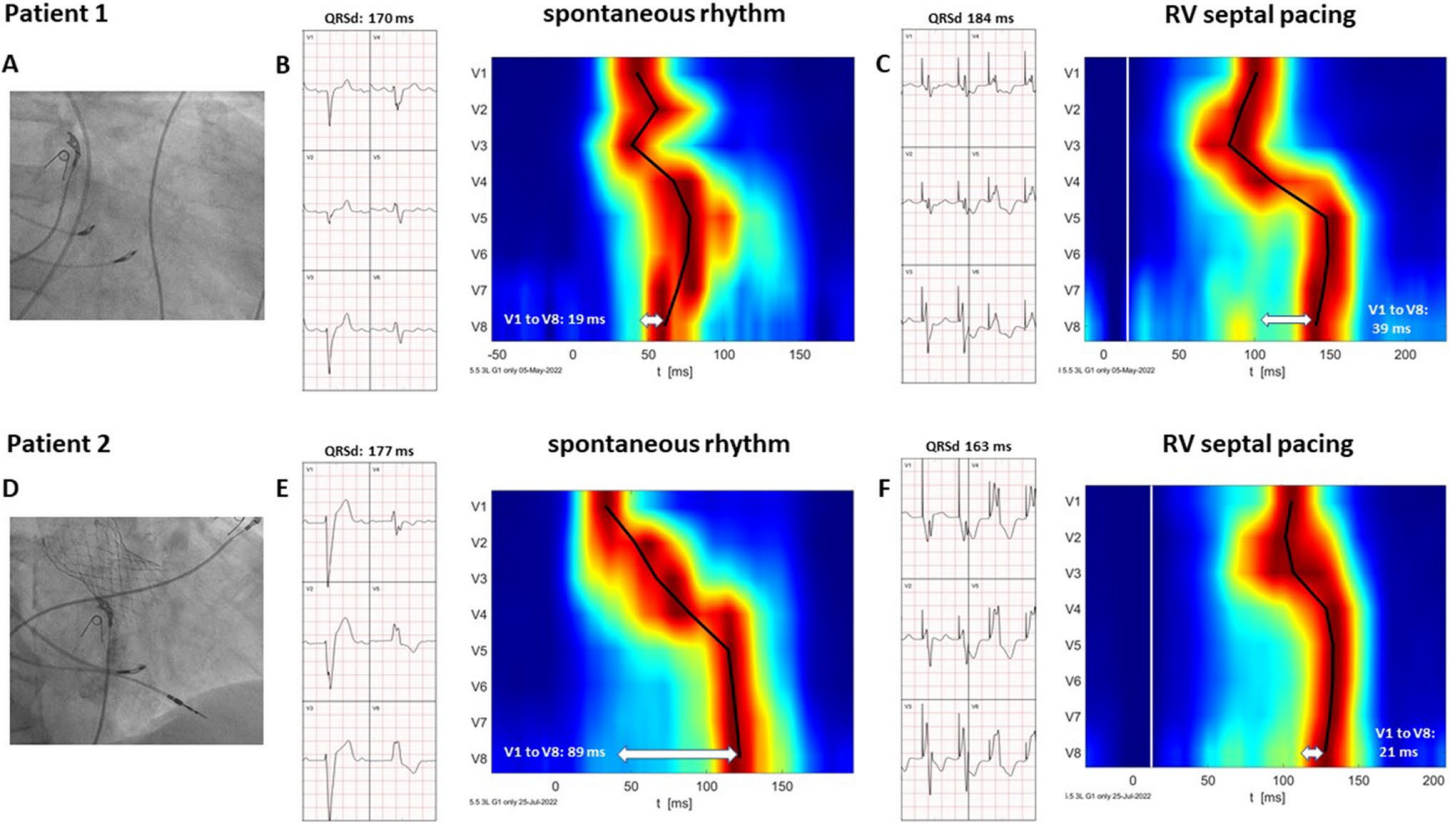
Visualization of the RVSP location using either His bundle location (**A**) or a septogram to visualize the tricuspid valve (**D**). Precordial lead ECGs and depolarization maps during spontaneous rhythm are shown in **B** and **E** and during RVSP in **C** and **F**, respectively. RVSP, right ventricular septal pacing

In patient 1, the delay between V1 and V8 activation was 19 ms (Fig. 1, panel B). In patient 2, the activation delay between V1 and V8 was 89 ms (Fig. 1, panel E). During RVSP, different changes in QRSd and V1 to V8 time delays were observed. In the first patient, QRSd increased from 170 to 184 ms, and the V1 to V8 activation delay increased from 19 to 39 ms (Fig. 1, panels B and C). In the second patient, QRSd shortened from 177 to 163 ms, and the V1 to V8 activation delay decreased from 89 to 21 ms (Fig. 1, panels E and F).

Studied patients differed in their UHF-ECG ventricular activation sequence during spontaneous rhythm and RVSP. The patient with IVCD presented with minimal interventricular dyssynchrony and the latest activation under V5. On the other hand, the patient with a trueLBBB had significant dyssynchrony and the latest activation under lead V8 during spontaneous rhythm. RVSP resulted in a different set of changes in ventricular synchrony. In patient 1, the interventricular dyssynchrony increased due to the additional trans-septal delay, which was absent during spontaneous rhythm. In patient 2, UHF-ECG interventricular dyssynchrony decreased during RVSP. This happened because, during pacing of the basal interventricular septum, the trans-septal and consequent LV activation started immediately after pacing; however, RV lateral wall activation was postponed by the time interval required for the electrical wave-front to reach the right bundle branch Purkinje fibers located in the distal part of the RV septum.

In patients with heart failure and wide QRS complexes on ECG, we demonstrated different ventricular activation patterns during a spontaneous rhythm and in response to RV septal pacing. Further research is needed to determine if the assessment of ventricular depolarization using UHF-ECG can discriminate between trueLBBB vs. IVCD or even those with mixed levels of block, i.e., a combination of trueLBBB and IVCD.

## Data Availability

The data supporting this study’s findings are available on request from the corresponding author. The data are not publicly available due to privacy or ethical restrictions.
